# Galectin-3 restrains T cell-driven inflammation in the testis and epididymis

**DOI:** 10.1038/s41419-026-09304-w

**Published:** 2026-09-26

**Authors:** Hiba Hasan, Kimberly Jandel, Eva Wahle, Gabriela Michel, Linda Kreyling, Vishnu Kumar, Tara Procida-Kowalski, Sudhanshu Bhushan, Michaela Frolikova, Jens Rosellen, Thorsten Diemer, Katerina Komrskova, Marek Bartkuhn, Martin Speckmann, Hans-Christian Schuppe, Daniela Fietz, Christiane Pleuger, Andreas Meinhardt, Monika Fijak

**Affiliations:** 1 Institute of Anatomy and Cell Biology, Justus Liebig University of Giessen, Giessen, Germany; 2 Hessian Centre of Reproductive Medicine, Justus Liebig University of Giessen, Giessen, Germany; 3 Institute for Clinical Immunology, Transfusion Medicine and Hemostaseology, JLU cDeeP Core Facility, Justus-Liebig-University Giessen, Giessen, Germany; 4 Biomedical Informatics and Systems Medicine Science Unit for Basic and Clinical Medicine, Justus Liebig University of Giessen, Giessen, Germany; 5 Laboratory of Reproductive Biology, Institute of Biotechnology of the Czech Academy of Sciences, Vestec, Czech Republic; 6 Department of Urology, Paediatric Urology and Andrology, Justus Liebig University of Giessen, Giessen, Germany; 7 Department of Zoology, Faculty of Science, Charles University, Prague, Czech Republic; 8 Department of Veterinary Anatomy, Histology and Embryology, Justus Liebig University of Giessen, Giessen, Germany; 9 Centre for Reproductive Health, Hudson Institute of Medical Research, Clayton, VIC Australia

**Keywords:** Chronic inflammation, Autoimmunity

## Abstract

Galectin-3 (Gal-3) is a pleiotropic molecule involved in immune regulation and tissue homeostasis. While it has been implicated in reproductive tissue function, its role in shaping immune cell dynamics within the testis and epididymis remains incompletely defined. Here, we investigated the function of Gal-3 in the testis and epididymis under steady-state and inflammatory conditions, to determine its contribution to inflammatory responses and tissue damage. In both murine and human tissues, Gal-3 frequency and staining intensity increased along the testicular-epididymal axis, with the highest proportion of Gal-3^+^ cells in the cauda epididymidis, where it was detected across multiple murine immune cell populations, as well as epithelial and germ cells. Consistent with this distribution, Gal-3 deficiency reshaped the local immune landscape, leading to reduced macrophage populations and increased T cell abundance in the testis and cauda epididymidis. In a model of experimental autoimmune epididymo-orchitis (EAEO), Gal-3–deficient (*Lgals3*^*-/-*^) mice exhibited exacerbated inflammation, fibrosis, and irreversible organ damage, accompanied by increased T cell frequencies among CD45^+^ cells. Transcriptomic analysis revealed enhanced T cell activation signatures, particularly involving IFN-γ–related pathways. Functionally, Gal-3-deficient T cells showed increased cytokine production, specifically IFN-γ, which was further amplified in the presence of Gal-3-deficient macrophages, which in turn acquired a pro-inflammatory phenotype. Together, these findings identify Gal-3 as a key regulator of T cell activity and macrophage polarization with prominent effects in the testis and epididymis, with implications for understanding immune-mediated infertility.

## Introduction

The testis is an immunoprivileged site in which auto-antigenic germ cells are protected from immune attack while preserving the capacity to mount effective immune responses against pathogens. This balance is maintained through a combination of structural, cellular, and molecular mechanisms that support immune tolerance without compromising immune competence [1, 2]. Following their development in the testis, germ cells transit through the epididymis, where they undergo structural, functional, and biochemical maturation required for fertilization [3, 4]. This coordinated system ensures tight regulation of immune homeostasis across distinct but functionally linked tissues of the male reproductive tract.

Disturbances in the mechanisms governing testicular and epididymal immune balance may predispose to infertility. Inflammation affecting the testis and epididymis, of non-infectious idiopathic origin, is a significant cause of infertility. 25-30% of men with azoospermia exhibit evidence of testicular inflammatory lesions, yet the etiopathogenesis remain not well understood [5, 6]. Experimental autoimmune epididymo-orchitis (EAEO) is a model of chronic testicular inflammation mimicking chronic pathological changes found in patient biopsies. The disease is characterized by immune cell infiltration, elevation of pro-inflammatory and pro-fibrotic mediators, germ cell damage and fibrotic remodeling leading subsequently to infertility [6–9]. While multiple factors and mediators have been implicated in maintaining local immune homeostasis, the extent to which specific regulators coordinate immune cell composition and function across tissues and under inflammatory conditions is unclear [10, 11]. Defining such regulators is essential for understanding how immune homeostasis is maintained and modulated in physiological and pathological contexts.

Galectin-3 (Gal-3) is an immunoregulatory lectin with context- and tissue-dependent functions, particularly in inflammatory and immune settings. Structurally, Gal-3 is a chimera-type β-galactoside-binding lectin containing a carbohydrate recognition domain and an N-terminal domain that enables oligomerization [12]. It is widely expressed and participates in diverse biological processes, including cell–cell interactions, matrix interactions, proliferation, differentiation, and apoptosis [12–14]. In immune contexts, Gal-3 has been shown to modulate cell activation, survival, and recruitment, with effects that vary depending on tissue and pathophysiological conditions supporting its immunoregulatory role [15–17]. In context of T cells, Gal-3 orchestrates the distribution of the cell surface glycoproteins, which can in turn shift T cells activities and lead to stronger T cell-driven responses in several autoimmune disease models as validated in mice lacking Gal-3 [14, 15, 18]. Furthermore, Gal-3 maintains macrophages in an anti-inflammatory immunosuppressive state, whereas its deletion usually shifts them towards a pro-inflammatory, more invasive phenotype [14, 19]. In inflammatory and autoimmune diseases, the role of Gal-3 is highly context-dependent, where in some cases it aggravates disease severity acting as a driver of pro-inflammatory phenotype of immune cells, while in others reduces tissue damage by suppressing immune cell activity [12, 13, 15, 16, 20]. Although previous reports have shown essential function of Gal-3 in testicular development, steroidogenesis and spermatogenesis [21], its role in regulating immune cell balance and inflammatory response in the testis and epididymis remains unexplored. Its expression in both the testis and epididymis suggests that it may contribute to the regulation of immune homeostasis within these organs [1, 21, 22]. Studies in mice have shown that Gal-3 deficiency delays the first wave of spermatogenesis, reduces germ cell numbers, and increases germ cell apoptosis, resulting in mild testicular atrophy [21]. In addition, *Lgals3*^*−/−*^ mice exhibit enhanced steroidogenesis and reduced numbers of testicular macrophages, pointing to a role in regulating the local immune environment [1, 21]. Given these essential reproductive, endocrine and immunoregulatory roles of Gal-3, understanding how it shapes immune cell composition and function within the testis and epididymis becomes fundamental, particularly in a tissue-specific context and under inflammatory conditions.

Here, we investigated the role of Gal-3 in regulating immune homeostasis in the testis and epididymis under steady-state conditions and during chronic inflammation. Using Gal-3-deficient mice, we examined how the absence of Gal-3 affects immune cell landscape and local immune responses across these tissues.

## Results

### Gal-3 exhibits region-specific expression across the mouse testis and epididymis

**Fig. 1:**
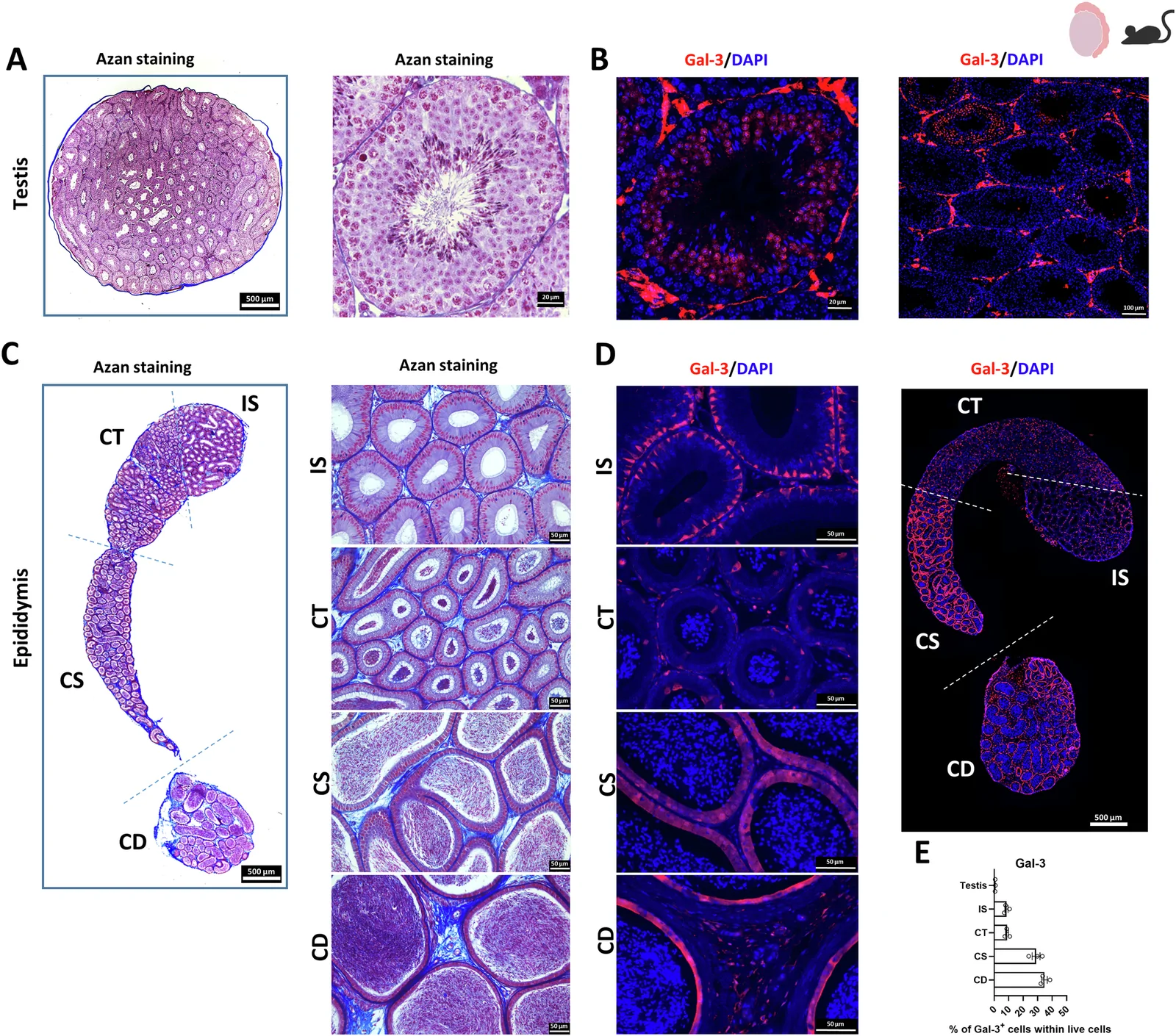
Gal-3 expression across the testis and epididymis. **A**, **C** Representative photomicrographs of Azan staining of paraffin sections from *WT* testis (**A**) and epididymis (**C**). **B**, **D** Immunofluorescence staining for Gal-3 (red) with nuclear DAPI (blue) in corresponding sections of WT testis (**B**) and epididymis (**D**). **E** Percentages of Gal-3–positive cells within live cells in testis, initial segment (IS), caput (CT), corpus (CS), and cauda (CD) epididymidis, determined by flow cytometry (*n* = 3).

To determine whether Gal-3 expression varies in the testis and epididymis, we first characterized its cellular distribution and regional expression in the adult murine testis and epididymis. In the testis, after verifying the specificity of Gal-3 antibody, Gal-3 expression was primarily restricted to interstitial cells and a subset of germ cells, while Sertoli cells were negative (Fig. 1A, B and Supplementary Fig. 1). By contrast, the epididymis exhibited a marked regional variation in Gal-3 expression, with both intensity and localization differing along the proximal–distal axis (Fig. 1C, D and Supplementary Fig. 1). In the proximal regions, including the initial segment (IS) and caput (CT), Gal-3 expression was relatively low and predominantly localized within epithelial cells, with only occasional interstitial positivity (Fig. 1C, D and Supplementary Fig. 1). In the distal regions, particularly the corpus (CS) and cauda (CD), Gal-3 expression was substantially increased. In these regions, the majority of epithelial cells were Gal-3 positive, with stronger staining intensity and additional nuclear localization (Fig. 1D and Supplementary Fig. 1). Notably, the CD also showed strong interstitial expression of Gal-3.

Quantification by flow cytometry confirmed these observations, demonstrating that the proportion of Gal-3-positive cells was lowest in the testis and progressively increased toward the distal epididymis, with the highest levels detected in the CD (Fig. 1E). These findings demonstrate that Gal-3 expression is highly region-specific along the epididymal tract, with a clear proximal-to-distal gradient and distinct cellular localization patterns, suggesting that Gal-3 may have context-dependent roles in different anatomical compartments.

### Gal-3 is preferentially expressed in immune, germ and epithelial cells in the testis and epididymis

**Fig. 2:**
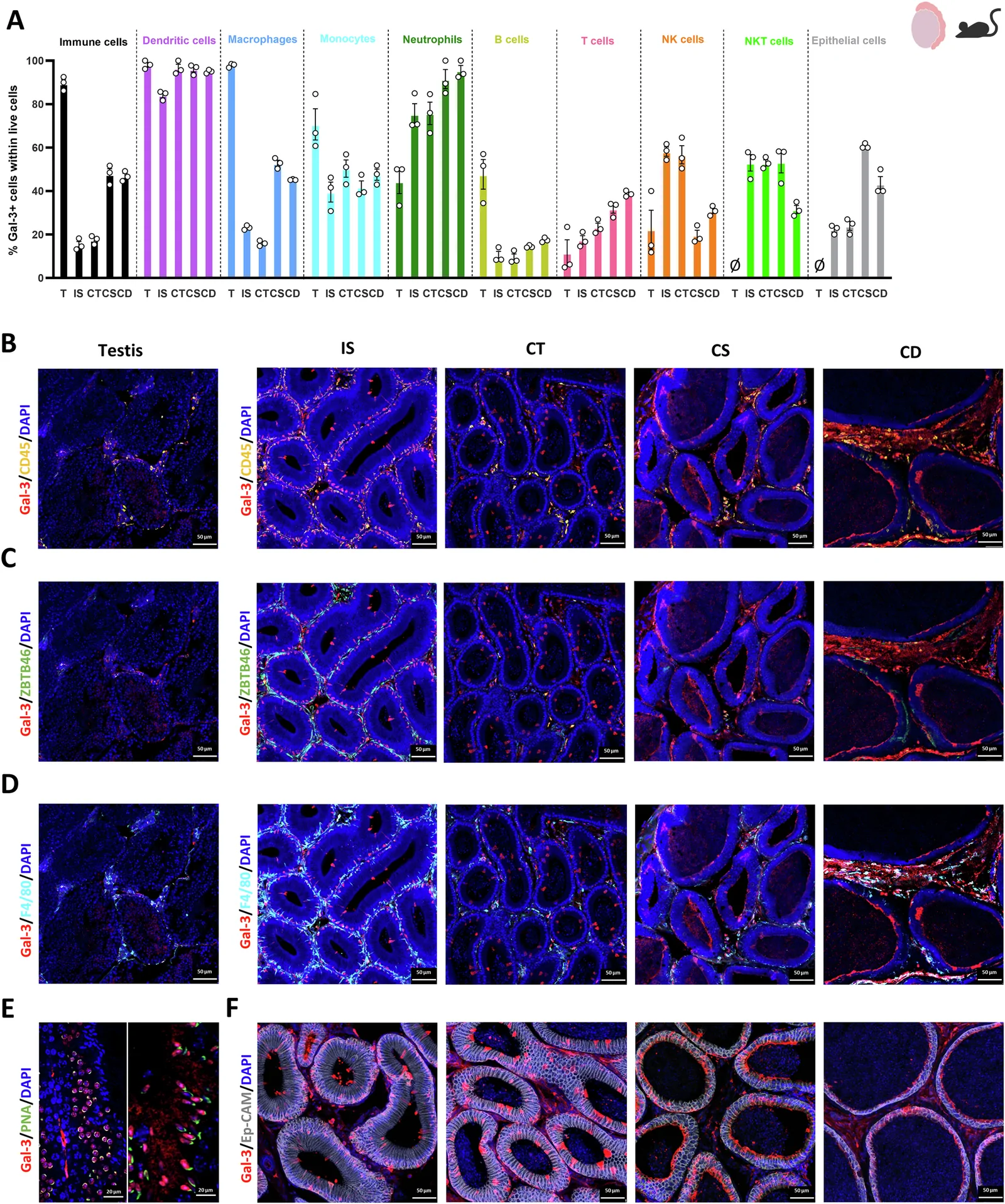
Gal-3 expression across immune cell populations in the testis and the epididymis. **A** Percentages of Gal-3^+^ cells within immune cells (CD45^+^), dendritic cells (CD11c^+^MHCII^+^), macrophages (F4/80^+^CD11b^+^), monocytes (Ly6c^+^), neutrophils (Ly6g^+^), B cells (CD19^+^), T cells (CD3^+^NK1.1^−^), NK cells (CD3^-^NK1.1^+^), NKT cells (CD3^+^NK1.1^+^) and epithelial cells (Ep-CAM^+^) in *WT* testis, IS, CT, CS, and CD epididymidis, determined by flow cytometry (*n* = 3). Ø, not examined. **B**–**D** Representative immunofluorescence images of Gal-3 (red), CD45 (yellow), and F4/80 (light blue) with nuclear DAPI (blue) in frozen sections from *Zbtb46*^GFP^ testis and epididymis. **E** Representative immunofluorescence images of Gal-3 (red) and PNA (green) with nuclear DAPI (blue) in paraffin sections from *WT* testis. **F** Representative immunofluorescence images of Gal-3 (red) and Ep-CAM (grey) with nuclear DAPI (blue) in frozen sections from *WT* epididymis.

Given the differential expression patterns of Gal-3 across the testis and epididymis, we next investigated its presence in major immune cell populations using immunofluorescence staining and high-dimensional flow cytometry (Supplementary Fig. 2). In the testis, more than 90% of immune cells were positive for Gal-3 (Fig. 2A–D and Supplementary Figs 3A, B). In the epididymis, this proportion was lower overall but increased toward the distal regions compared with the proximal segments (Fig. 2A, B and Supplementary Fig. 4A).

Across both tissues, cells of the myeloid lineage (dendritic cells, macrophages, monocytes, and neutrophils) exhibited higher Gal-3 expression than lymphoid populations (B cells, T cells, NK cells, and NKT cells) (Fig. 2A). Dendritic cells were almost uniformly positive for Gal-3 in both the testis and epididymis, although slightly lower proportions were observed in the IS (Fig. 2A, C and Supplementary Figs. 3B and 4A). By contrast, macrophages and monocytes showed the highest levels of Gal-3 expression in the testis (Fig. 2A, D and Supplementary Fig. 3A, B).

Within the epididymis, macrophages displayed a gradient-like increase in Gal-3 expression from proximal to distal regions, whereas this pattern was not evident in monocytes (Fig. 2A, D and Supplementary Fig. 4A). Neutrophils and T cells similarly exhibited increasing Gal-3 expression toward the CD, while B cells showed higher expression in the testis compared with the epididymis (Fig. 2A). In addition to immune cells, Gal-3 expression was detected in later stages of germ cell development, including early round spermatids, late spermatids, and mature spermatozoa, as indicated by acrosomal co-localization with PNA (Fig. 2E and Supplementary Fig. 3A, C). Among epithelial cells, the highest proportion of Gal-3-positive cells was observed in the CS followed by the CD (Fig. 2A, F and Supplementary Fig. 4B).

Together, these findings demonstrate that Gal-3 expression is enriched across different immune cell populations and exhibits cell type- and region-specific variation in the testis and epididymis, further supporting a context-dependent role in local immune regulation.

### Gal-3 deficiency alters the immune cell composition in the testis and epididymis

**Fig. 3:**
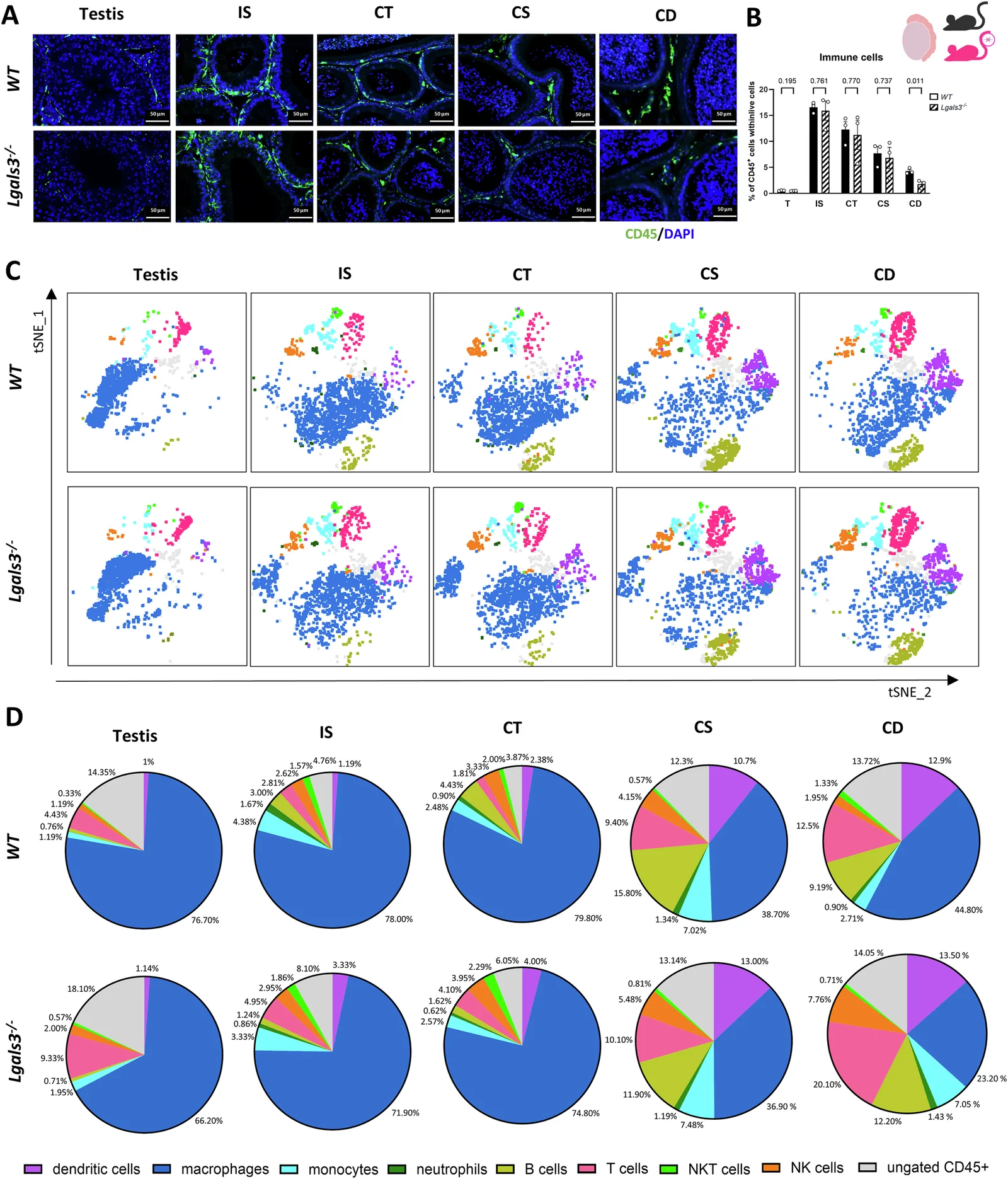
Immune cell landscape in the testis and epididymis in the absence of Gal-3. **A** Representative immunofluorescence images of CD45 (green) with nuclear DAPI (blue) in paraffin sections from *WT* and *Lgals3*^*−/−*^ testis and epididymis. **B** Percentages of CD45^+^ cells within live cells in *WT* and *Lgals3*^*−/−*^ testis, IS, CT, CS, and CD epididymidis, determined by flow cytometry (*n* = 3, mean ± SEM). Statistical analysis was performed using multiple unpaired *t* tests. **C** t-SNE (t-distributed stochastic neighbor embedding) plots generated from downsampled (700 CD45^+^ cells per sample) concatenated samples from *WT* and *Lgals3*^*−/−*^ testis, IS, CT, CS, and CD epididymidis. Cells were gated as shown in Supplementary Fig. 2. Samples from all biological groups (three biological replicates) were concatenated. **D** Pie charts showing the proportion of dendritic cells (CD11c^+^MHCII^+^), macrophages (F4/80^+^CD11b^+^), monocytes (Ly6c^+^), neutrophils (Ly6g^+^), B cells (CD19^+^), T cells (CD3^+^NK1.1^-^), NK cells (CD3^-^NK1.1^+^) and NKT cells (CD3^+^NK1.1^+^) from t-SNE analysis in *WT* and *Lgals3*^*−/−*^ testis, IS, CT, CS, and CD epididymidis.

Given the distinct expression patterns of Gal-3 across the testis and epididymis, we next investigated the impact of Gal-3 deficiency on tissue architecture and immune cell composition using *Lgals3*^*−/−*^ mice. Histological analysis revealed no major morphological differences between *Lgals3*^*−/−*^ and *WT* tissues, with preserved epithelial structure, tubular organization, and interstitial architecture in the testis and epididymis (Supplementary Fig. 5A). We therefore focused on the immune compartment. In *WT* animals, immune cell abundance decreased progressively from the proximal to distal epididymis and was lowest in the testis, consistent with previous reports [23–25] (Fig. 3A, B). Interestingly, in the absence of Gal-3, we observed a significant reduction in total immune cells, specifically in the CD, while other regions remained largely unaffected (Fig. 3B).

**Fig. 4:**
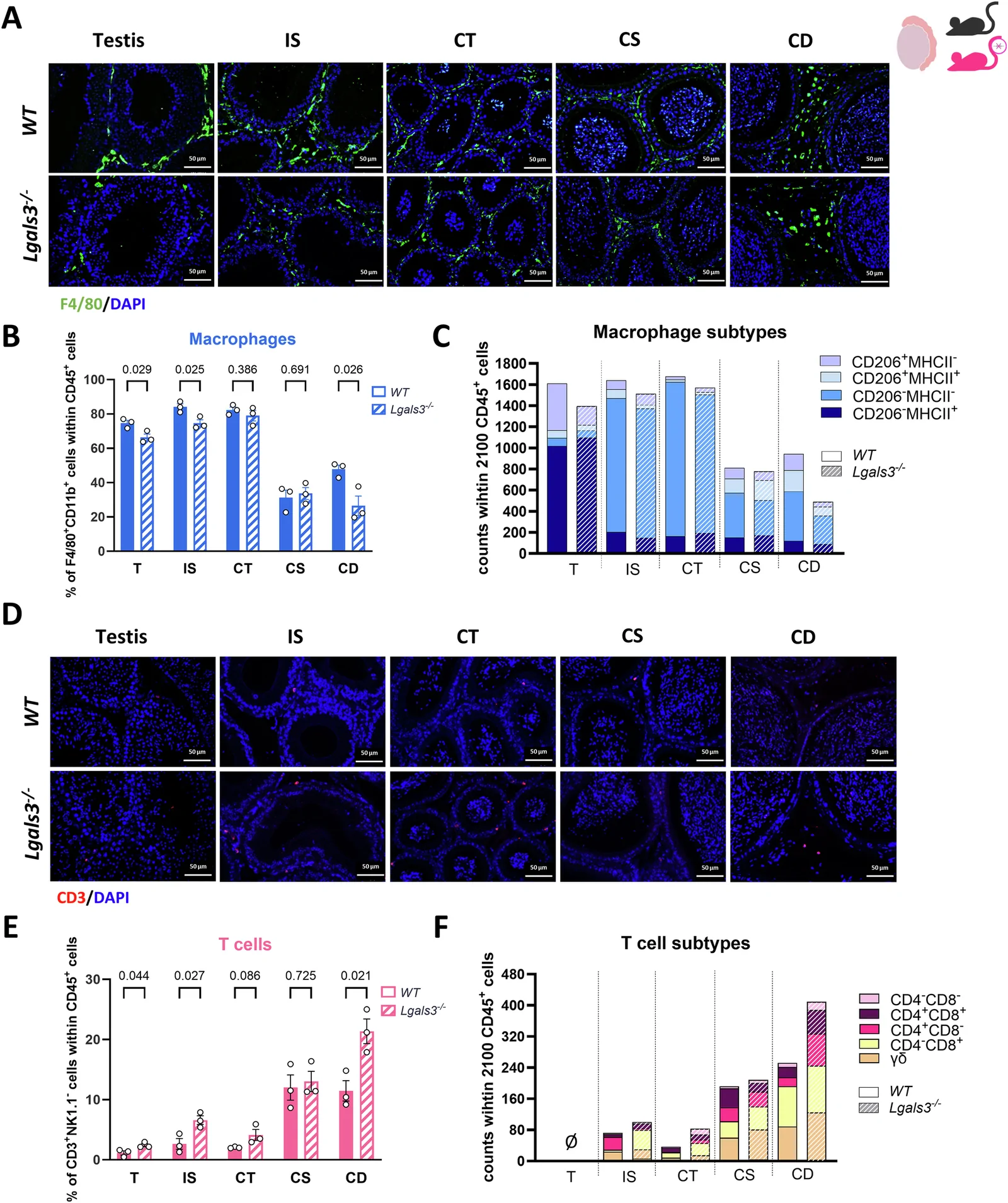
Macrophages and T cell populations in Gal-3 deficient testis and epididymis. **A**, **D** Representative immunofluorescence images of F4/80 (green) (**A**), CD3 (red) (**D**), and nuclear DAPI (blue) in paraffin sections from *WT* and *Lgals3*^*−/−*^ testis, IS, CT, CS, and CD epididymidis. **B**, **E** Percentages of macrophages (F4/80^+^CD11b^+^) (**B**) and T cells (CD3^+^NK1.1^−^) (**E**) within CD45^+^ cells in *WT* and *Lgals3*^*−/−*^ testis, IS, CT, CS, and CD epididymidis, determined by flow cytometry (*n* = 3, mean ± SEM). Statistical analysis was performed using multiple unpaired *t* tests. **C**, **F** Macrophage (**C**) and T cell (**F**) counts derived from concatenated samples displayed in t-SNE plots from *WT* and *Lgals3*^*−/−*^ testis, IS, CT, CS, and CD epididymidis (three biological replicates grouped).

To assess changes in immune cell composition, we analyzed major immune cell populations using t-SNE-based clustering of downsampled immune cells (Fig. 3C, D). Under steady-state conditions, macrophage proportions decreased toward the distal epididymis, whereas dendritic cell and T cell populations increased along this axis (Fig. 3C, D). Notably, Gal-3 deficiency altered this balance, with a marked reduction in macrophage proportions in the testis, IS, and CD, accompanied by a relative increase in T cell populations in the same regions (Fig. 4A–E). Other immune cell populations were largely unchanged, with the exception of a decrease in NKT cells in the CD (Fig. 3C, D and Supplementary Fig. 5B).

These findings indicate that Gal-3 contributes to maintaining immune cell composition in the testis and epididymis, with region-specific effects that are most pronounced in macrophage and T cell populations.

### Gal-3 deficiency reshapes macrophage and T cell subpopulations in a region-specific manner

Building on the observed changes in overall immune cell composition, we next examined whether Gal-3 deficiency affects specific immune cell subpopulations within the testis and epididymis. Based on CD206 and MHCII expression, macrophages were further stratified into four different populations. In the testis and CD of *Lgals3*^−/−^ mice, the proportion of CD206^+^MHCII^-^ macrophages was reduced, while CD206^-^MHCII^+^ macrophages were increased, compared to *WT* mice. In addition, the CD206^+^MHCII^+^ population was significantly decreased in the testis (Fig. 4C and Supplementary Fig. 5C). These results indicate a shift in the phenotypic composition of testis resident macrophage populations in the absence of Gal-3. We next assessed T cell subsets by classifying them according to the expression of alpha - beta (αβ) and gamma - delta (γδ) T cell receptor. αβ^+^ T cells were further divided based on the expression of their co-receptors CD4 and CD8. We found a significant increase in CD4^+^CD8^−^CD3^+^ cells specifically in the CD of *Lgals3*^−/−^ mice (Fig. 4F and Supplementary Fig. 5D), while other T cell subsets across epididymal regions were unaffected. Thus, Gal-3 deficiency not only alters overall immune cell composition but also reshapes macrophage and T cell subpopulations in a region-specific manner, with the most pronounced effects observed in the distal epididymis.

### Gal-3 regulates immune and tissue responses during testicular inflammation

Given the observed role of Gal-3 in shaping immune cell composition under steady-state conditions, we next sought to determine whether Gal-3 similarly modulates immune responses during inflammation. To address this, we induced experimental autoimmune epididymo-orchitis (EAEO) in *WT* and *Lgals3*^*−/−*^ mice as a model of testicular inflammation. EAEO is characterized by immune cell infiltration, disruption of seminiferous tubule architecture, germ cell loss, and fibrosis (Supplementary Fig. 6A).

**Fig. 5:**
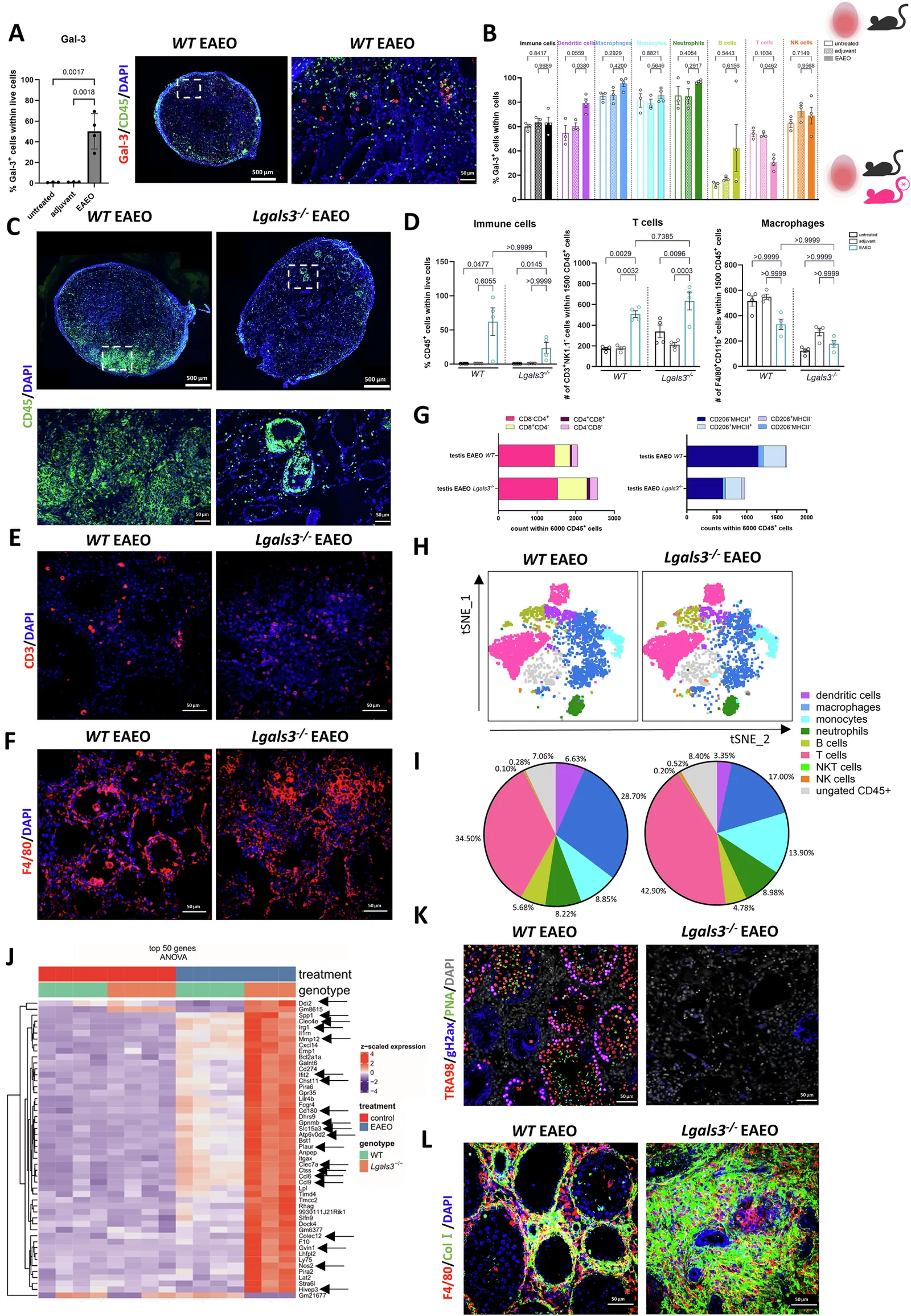
Testicular inflammation and tissue alterations in *Lgals3*^−/−^ mice during EAEO. **A** Percentages of Gal-3^+^ cells within live cells in untreated, adjuvant, and EAEO *WT* testis, determined by flow cytometry [*n* = 3 (untreated and adjuvant control; *n* = 4 (EAEO); mean ± SEM]. Statistical analysis was performed using Kruskal–Wallis tests with Dunn’s post-hoc comparisons. Representative immunofluorescence images of Gal-3 (red), CD45 (green), and nuclear DAPI (blue) in paraffin sections from *WT* EAEO testis. **B** Percentages of Gal-3⁺ cells within dendritic cells (F4/80^-^MHCII^+^), macrophages (F4/80^+^CD11b^+^), monocytes (Ly6c^+^), neutrophils (Ly6g^+^), B cells (CD19^+^), T cells (CD3^+^NK1.1^-^), and NK cells (CD3^-^NK1.1^+^) in untreated, adjuvant, and EAEO *WT* testis, determined by flow cytometry [*n* = 3 (untreated and adjuvant control; *n* = 4 (EAEO); mean ± SEM]. Statistical analysis was performed using one-way ANOVA with Tukey’s post-hoc test or Kruskal–Wallis tests with Dunn’s post-hoc comparisons. **C**, **E**, **F** Representative immunofluorescence images of CD45 (green) (**C**), CD3 (red) (**E**), and F4/80 (red) (**F**) with nuclear DAPI (blue) in paraffin sections from *WT and Lgals3*^*−/−*^ EAEO testis. **D** Percentages of immune cells (CD45^+^) within live cells, and counts of T cells (CD3^+^NK1.1^−^) and macrophages (F4/80^+^CD11b^+^) within CD45^+^ cells in untreated, adjuvant, and EAEO *WT* and *Lgals3*^*−/−*^ testis, determined by flow cytometry (*n* = 4, mean ± SEM). Statistical analysis was performed using two-way ANOVA with Tukey’s post-hoc test or Kruskal–Wallis tests with Dunn’s post-hoc comparisons. **G** Counts of T cell and macrophage subpopulations derived from concatenated, downsampled samples (1500 CD45^+^ cells per replicate) displayed in t-SNE plots from EAEO *WT* and *Lgals3*^*−/−*^ testis (four biological replicates grouped). **H** t-SNE (t-distributed stochastic neighbor embedding) plots generated from downsampled (1500 CD45^+^ cells per sample) concatenated samples from EAEO *WT* and *Lgals3*^*−/−*^ testis. Cells were gated as shown in Supplementary Fig. 2. Samples from all biological groups (four biological replicates) were concatenated. **I** Pie charts showing the proportion of dendritic cells (F4/80^-^MHCII^+^), macrophages (F4/80^+^CD11b^+^), monocytes (Ly6c^+^), neutrophils (Ly6g^+^), B cells (CD19^+^), T cells (CD3^+^NK1.1^-^), NK cells (CD3^-^NK1.1^+^), and NKT cells (CD3^+^NK1.1^+^) derived from t-SNE analysis in EAEO *WT* and *Lgals3*^*−/−*^ testis. **J** Heatmap of z-transformed expression values for the top 50 upregulated genes (ranked by *P* value) in EAEO *Lgals3*^*−/−*^ versus *WT* testis. **K** Representative immunofluorescence images of TRA98 (red), γH2AX (blue), PNA (green), and nuclear DAPI (blue) in paraffin sections from *WT* and *Lgals3*^*−/−*^ EAEO testis. **L** Representative immunofluorescence images of F4/80 (red), collagen I (Col I, green), and nuclear DAPI (blue) in paraffin sections from *WT* and *Lgals3*^*−/−*^ EAEO testis.

Under inflammatory conditions in *WT* mice, Gal-3 expression was significantly increased in live cells, but not within the immune cell compartment (Fig. 5A, B). In contrast to steady-state conditions, Gal-3-positive immune cells were now observed within damaged seminiferous tubules and in the interstitial space of inflamed tissue (Fig. 5A). At the cellular level, Gal-3 expression was increased in dendritic cells, remained unchanged in macrophages, but was reduced in T cells following EAEO induction (Fig. 5B).

We next examined how Gal-3 deficiency influences immune cell composition during inflammation. EAEO induction led to a marked increase in CD45^+^ cells in both genotypes; however, this increase was less pronounced in *Lgals3*^*−/−*^ mice compared with *WT* (Fig. 5C, D and Supplementary Fig. 6A). T cells, particularly CD4^+^ T cells, were significantly increased following EAEO in both groups, with a more pronounced expansion in the absence of Gal-3 (Fig. 5D, E, G and Supplementary Figs. 6B and 7A). While overall macrophage proportions were not significantly altered, the CD206^-^MHCII^+^ subset was reduced in *Lgals3*^*−/−*^ mice compared with *WT* under EAEO conditions (Fig. 5D, F, G and Supplementary Figs. 6C and 7B). In addition, CD8^+^ T cells and specific monocyte populations (CCR2^+^MHCII^−^ and CCR2^−^MHCII^−^) were increased following EAEO only in *Lgals3*^*−/−*^ mice (Fig. 5G–I and Supplementary Figs. 6D and 7C–E).

To further characterize the impact of Gal-3 deficiency, we performed transcriptomic analysis of EAEO testis. Among the top differentially expressed genes between *Lgals3*^*−/−*^ and *WT* mice, upregulated pathways were associated with stress and danger responses, (e.g., *Ddi2, Hivep3, Cd180, Clec7a*, *Clec4a*), interferon signaling (e.g., *Nos2, Gvin1, Slfn9, Ifit2, Irg1*), antigen presentation (e.g., *Ly75, Atp6v0d2, Slc15a3, Ctss*) and fibrotic remodeling (e.g., *Chst11, Gpnmb, Plaur, Mmp12*) (Fig. 5J and Supplementary Fig. 8). Genes linked to cell death were also elevated in the absence of Gal-3 (Supplementary Fig. 8).

Consistent with these transcriptional changes, *Lgals3*^*−/−*^ EAEO testis exhibited more pronounced tissue damage, including germ cell loss and increased damage within seminiferous tubules (Fig. 5K and Supplementary Fig. 6E, F). In addition, enhanced fibrotic remodeling was observed, as indicated by increased collagen deposition (Fig. 5L and Supplementary Fig. 6A, G).

These results imply that Gal-3 modulates immune responses and tissue integrity during testicular inflammation, with its absence associated with exacerbated immune activation and tissue damage.

### Gal-3 deficiency alters immune cell composition in the cauda epididymidis during EAEO

**Fig. 6:**
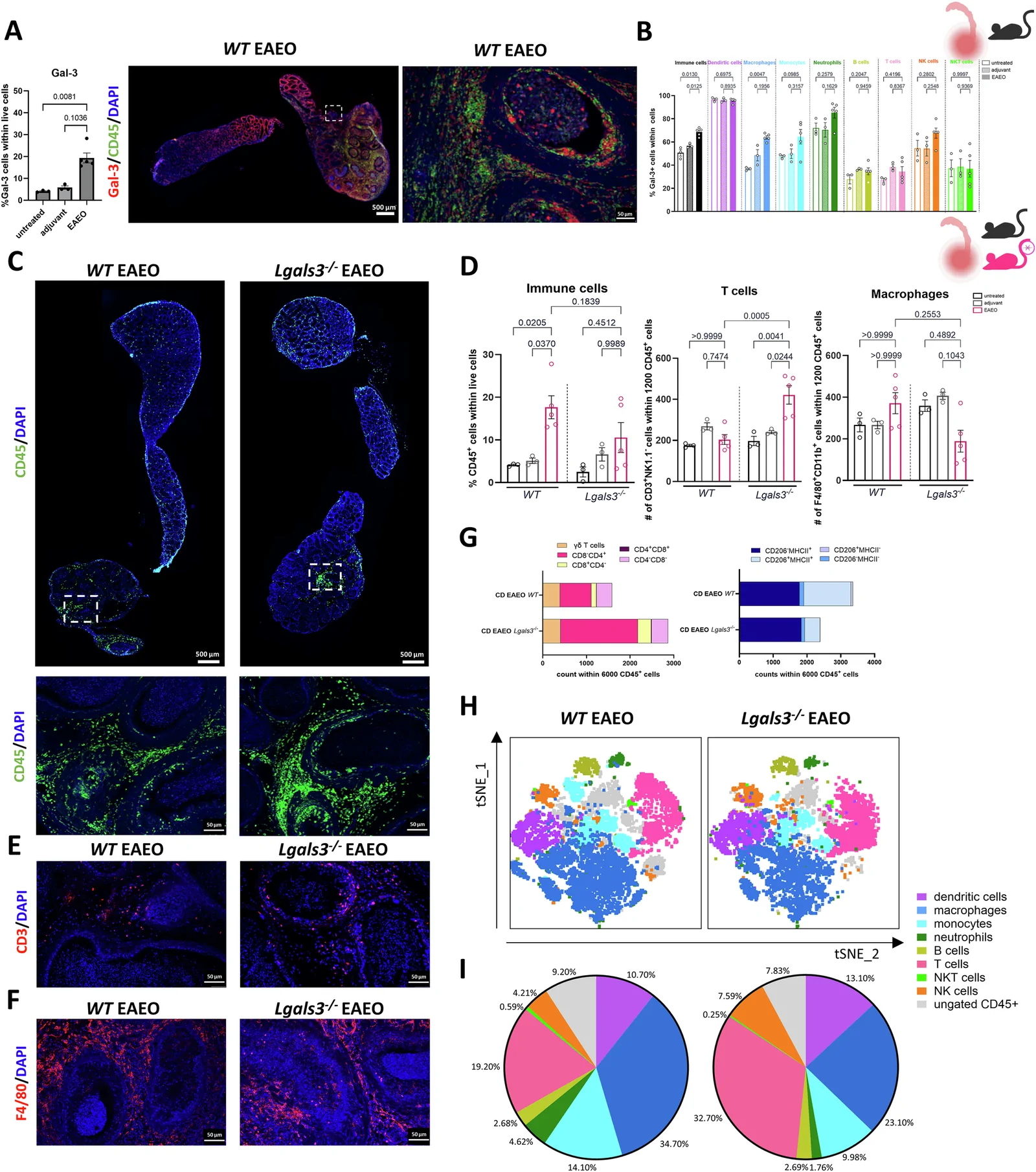
Immune cell composition in the cauda epididymidis of *Lgals3*^−/−^ mice during EAEO. **A** Percentages of Gal-3^+^ cells within live cells in untreated, adjuvant, and EAEO *WT* CD epididymidis, determined by flow cytometry [*n* = 3 (untreated and adjuvant control; *n* = 5 (EAEO); mean ± SEM]. Statistical analysis was performed using one-way ANOVA with Tukey’s post-hoc test. Representative immunofluorescence images of Gal-3 (red), CD45 (green), and nuclear DAPI (blue) in paraffin sections from *WT* EAEO CD epididymidis. **B** Percentages of Gal-3⁺ cells within dendritic cells (CD11c^+^MHCII^+^), macrophages (F4/80^+^CD11b^+^), monocytes (Ly6c^+^), neutrophils (Ly6g^+^), B cells (CD19^+^), T cells (CD3^+^NK1.1^-^), NK cells (CD3^-^NK1.1^+^), and NKT cells (CD3^+^NK1.1^+^) in untreated, adjuvant, and EAEO *WT* CD epididymidis, determined by flow cytometry [*n* = 3 (untreated and adjuvant control; *n* = 5 (EAEO); mean ± SEM]. Statistical analysis was performed using one-way ANOVA with Tukey’s post-hoc test or Kruskal–Wallis tests with Dunn’s post-hoc comparisons. **C**, **E**, **F** Representative immunofluorescence images of CD45 (green) (**C**), CD3 (red) (**E**), and F4/80 (red) (**F**) with nuclear DAPI (blue) in paraffin sections from *WT and Lgals3*^*−/−*^ EAEO CD epididymidis. **D** Percentages of immune cells (CD45^+^) within live cells, and counts of T cells (CD3^+^NK1.1^−^) and macrophages (F4/80^+^CD11b^+^) within CD45^+^ cells in untreated, adjuvant, and EAEO *WT* and *Lgals3*^*−/−*^ CD epididymidis, determined by flow cytometry [*n* = 3 (untreated and adjuvant control), *n* = 5 (EAEO); mean ± SEM]. Statistical analysis was performed using two-way ANOVA with Tukey’s post-hoc test or Kruskal–Wallis tests with Dunn’s post-hoc comparisons. **G** Counts of T cell and macrophage subpopulations derived from concatenated, downsampled samples (1200 CD45⁺ cells per replicate) displayed in t-SNE plots from EAEO *WT* and *Lgals3*^−/−^ CD epididymidis (five biological replicates grouped). **H** t-SNE (t-distributed stochastic neighbor embedding) plots generated from downsampled (1200 CD45^+^ cells per sample) concatenated samples from EAEO *WT* and *Lgals3*^*−/−*^ CD epididymidis. Cells were gated as shown in Supplementary Fig. 2. Samples from all biological groups (five biological replicates) were concatenated. **I** Pie charts showing the proportion of dendritic cells (CD11c^+^MHCII^+^), macrophages (F4/80^+^CD11b^+^), monocytes (Ly6c^+^), neutrophils (Ly6g^+^), B cells (CD19^+^), T cells (CD3^+^NK1.1^−^), NK cells (CD3^−^NK1.1^+^), and NKT cells (CD3^+^NK1.1^+^) derived from t-SNE analysis in EAEO *WT* and *Lgals3*^*−/−*^ CD epididymidis.

Given the region-specific effects observed during EAEO, we next focused on the CD, which is particularly susceptible to inflammatory responses (Supplementary Fig. 9A) [1, 11]. Using high-dimensional flow cytometry, we assessed Gal-3 expression and immune cell composition in the EAEO CD. Under inflammatory conditions, the number of live cells and CD45^+^ cells expressing Gal-3 was significantly increased in the CD compared with controls (Fig. 6A–D). In both genotypes, immune cell clusters were prominently detected in the EAEO CD, whereas such clustering was not observed in other epididymal regions (Fig. 6C).

In *WT* mice, Gal-3 expression was significantly increased in macrophages under EAEO conditions compared with control (Fig. 6B). Although total CD45^+^ cell numbers were significantly elevated only in WT EAEO, T cells (particularly CD4⁺ T cells) were significantly increased only in *Lgals3*^*−/−*^ EAEO CD (Fig. 6D, E, G and Supplementary Figs. 9B and 10A).

Interestingly, in *Lgals3*^*−/−*^ EAEO CD, both CD206^+^MHCII^+^ and CD206^+^MHCII^−^ were the major decreased macrophages subsets (Fig. 6D, F, G and Supplementary Figs. 9C and 10B). Other caudal immune cell populations were not changed in the absence of Gal-3 during EAEO (Fig. 6H, I and Supplementary Figs. 9D and 10C–E). Together, these findings indicate that Gal-3 deficiency is associated with a shift in immune cell composition, specifically macrophages and T cells, in the CD during inflammation, characterized by increased CD4⁺ T cells and altered macrophage subpopulations.

### Gal-3 deficiency enhances T cell cytokine responses and promotes pro-inflammatory macrophage polarization in co-culture

Given the increased T cell abundance observed under steady-state and inflammatory conditions in the absence of Gal-3, we next investigated whether Gal-3 also influences T cell functional activity. To this end, we assessed cytokine production by T cells lacking Gal-3 and examined how these responses are modulated in the presence of macrophages.

**Fig. 7:**
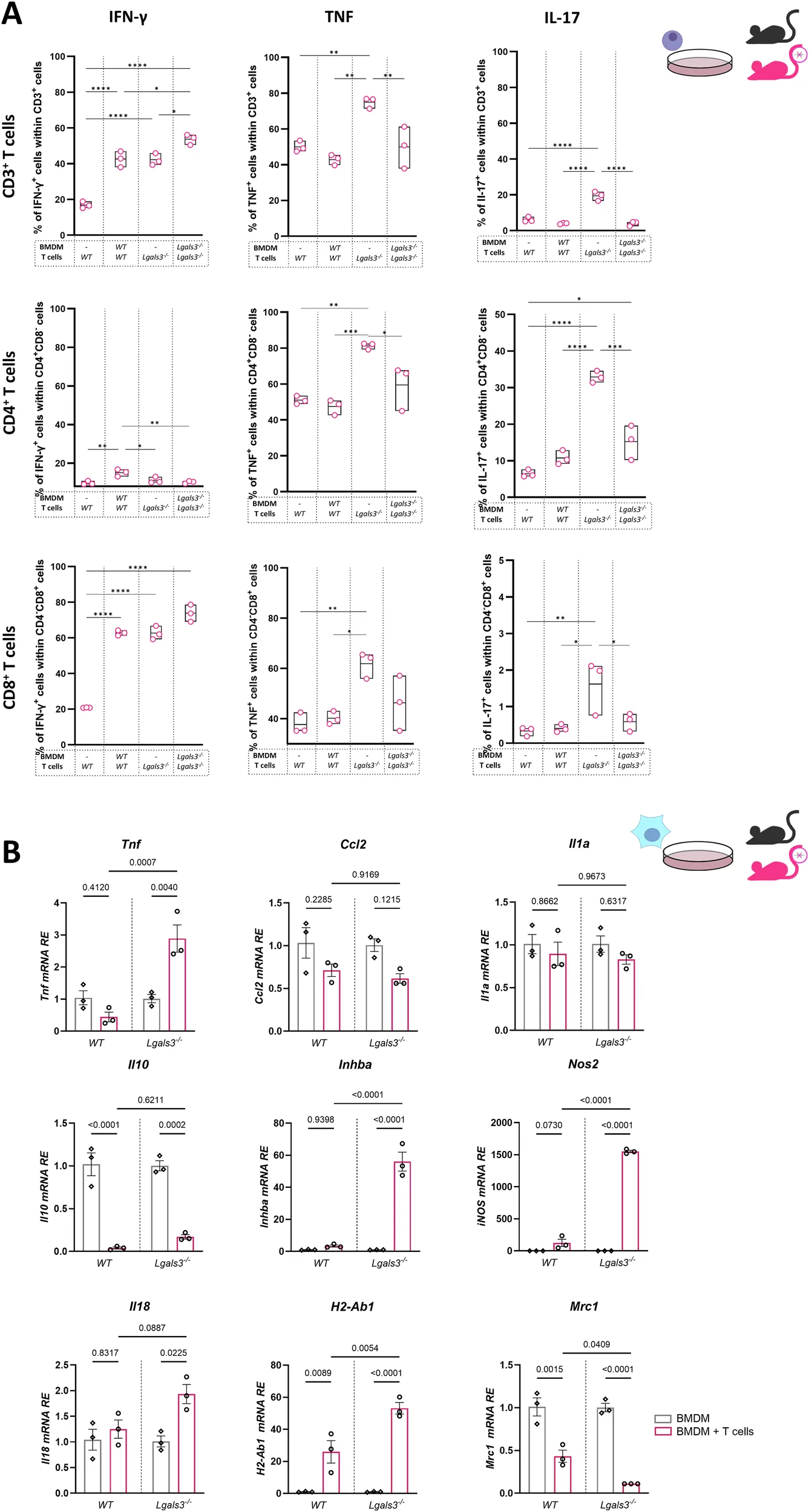
Cytokine production in T cells and macrophages profile in co-culture. **A** Percentages of IFN-γ^+^, TNF^+^, and IL-17A^+^ cells in *WT* or *Lgals3*^*−/−*^ CD3^+^, CD3^+^CD4^+^CD8^-^, and CD3^+^CD4^−^CD8^+^ T cell populations, determined by flow cytometry following in vitro culture alone or co-culture with *WT* or *Lgals3*^*−/−*^ BMDMs (*n* = 3, mean ± SEM). Statistical analysis was performed using one-way ANOVA with Tukey’s post-hoc test. **B** Relative expression (RE) of *Tnf*, *Ccl2, Il1a, Il10, Inhba, Nos2, Il18, H2-Ab1*, *and Mrc1* in *WT* and *Lgals3*^−/−^ BMDMs co-cultured with *WT* or *Lgals3*^−/−^ T cells, measured by quantitative real-time PCR. Expression was normalized to hypoxanthine–guanine phosphoribosyltransferase (*Hprt*) (*n* = 3, mean ± SEM). Statistical analysis was performed using two-way ANOVA with Tukey’s post-hoc test.

Splenic CD3^+^ T cells isolated from *WT* and *Lgals3*^*−/−*^ mice were cultured alone or in combination with *WT* and/or *Lgals3*^*−/−*^ BMDMs. T cells from *Lgals3*^*−/−*^ mice showed significantly increased production of IFN-γ, TNF, and IL-17 compared with *WT* controls (Fig. 7A). This increase was also observed in both CD4^+^ and CD8^+^ T cell subsets, with the exception of IFN-γ in CD4^+^ T cells, which remained unchanged (Fig. 7A and Supplementary Fig. 11A).

Co-culture with *WT* BMDMs enhanced IFN-γ production by *WT* CD3^+^ T cells and their subsets. In contrast, co-culture of *Lgals3*^*−/−*^ T cells with *Lgals3*^*−/−*^ BMDMs further increased IFN-γ production specifically in CD8⁺ T cells^+^, but not CD4^+^ subset, while TNF and IL-17 levels were reduced in both CD4⁺ and CD8⁺ subsets (Fig. 7A). Interestingly, the effect of *Lgals3*^*−/−*^ BMDMs on T cell responses was independent of T cell origin, as both *WT*- and *Lgals3*^*−/−*^*-*derived T cells exhibited similar cytokine patterns in their presence (Supplementary Fig. 11B).

CD8⁺ T cells exhibited higher IFN-γ production compared with CD4⁺ T cells (Fig. 7A). Consistent with this pattern, Gal-3 expression was higher in CD8⁺ T cells, particularly in the testis (Supplementary Fig. 11C). In the presence of *Lgals3*^*−/−*^ T cells, macrophages displayed increased expression of pro-inflammatory genes, including *Tnf, Inhba, Nos2, H2-Ab1*, and *Il18*, alongside reduced expression of *Mrc1* (Fig. 7B). Consistent with these in vitro findings, genes associated with T cell activation and IFN-γ signaling were elevated in *Lgals3*^*−/−*^ EAEO testis (Supplementary Figs. 8D–F). Together, these findings indicate that Gal-3 deficiency is associated with enhanced T cell cytokine responses and a shift toward a pro-inflammatory macrophage phenotype.

### Gal-3 displays a conserved expression pattern in human testis and cauda epididymidis and is upregulated in inflamed human testis

**Fig. 8:**
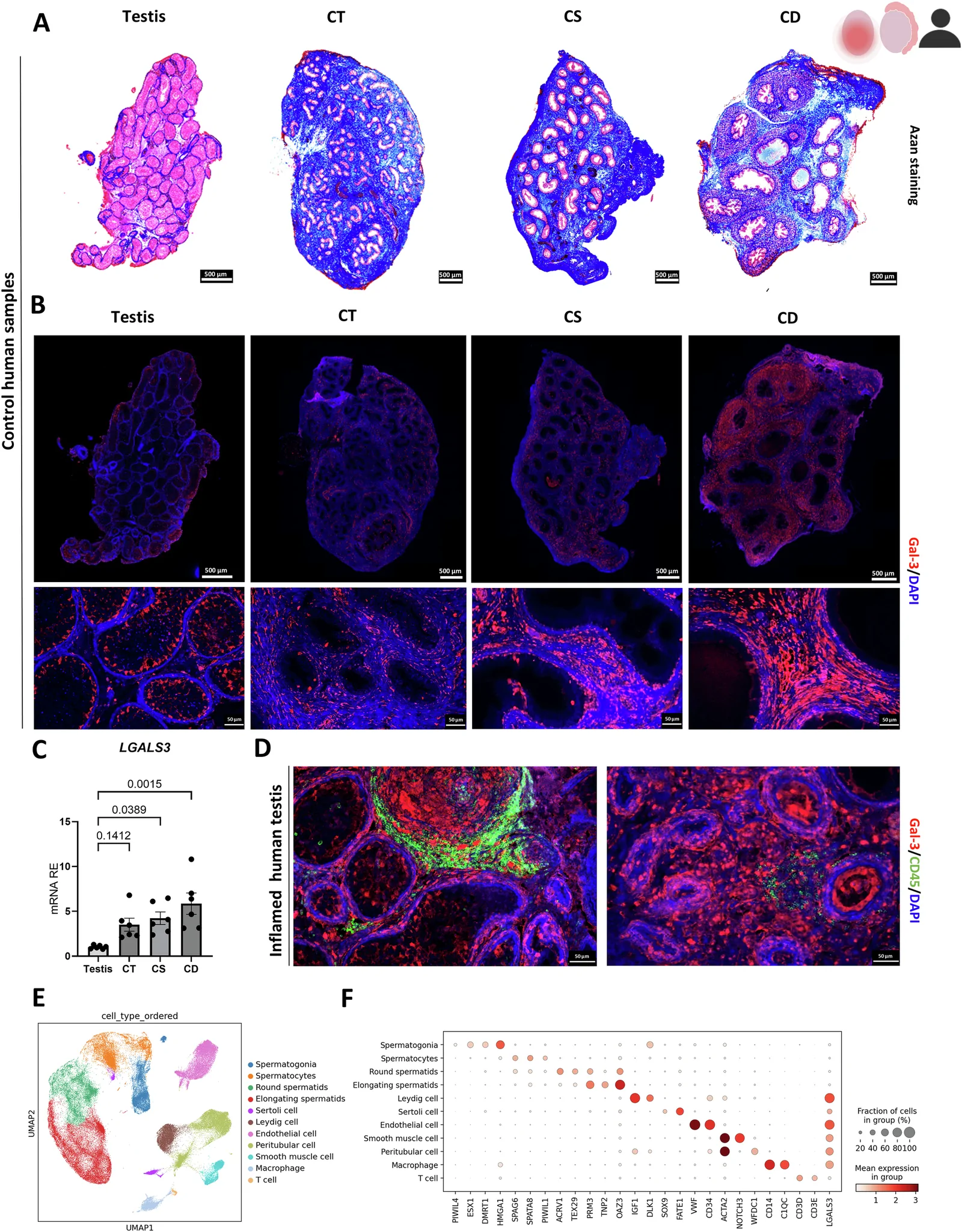
Gal-3 expression across the human testis and epididymis under normal and inflammatory conditions. **A** Representative Azan staining of paraffin sections from control human testis, CT, CS, and CD epididymides. **B** Representative immunofluorescence images of Gal-3 (red) and nuclear DAPI (blue) in corresponding sections. **C** Relative expression (RE) of *LGALS3* in control human testis, CT, CS, and CD epididymides, measured by quantitative real-time PCR. Expression was normalized to ribosomal protein S27 (*RPS27*) and ribosomal protein L19 (*RPL19*) (*n* = 6, mean ± SEM). Statistical analysis was performed using one-way ANOVA with Tukey’s post-hoc test. **D** Representative immunofluorescence images showing Gal-3 (red), CD45 (green), and nuclear DAPI (blue) in paraffin sections of inflamed testis from human patients with impaired spermatogenesis and focal leukocytic infiltrates. **E** Single-cell transcriptomic analysis of healthy human adult testes based on data retrieved from publicly available repositories. UMAP visualization of annotated testicular cell types from healthy donors (*n* = 26). Each dot represents a single testicular cell and is colored according to its assigned cellular cluster. **F** Dot plot displaying marker gene expression for each annotated testicular cell type together with *LGALS3* expression. Dot size indicates the percentage of cells expressing each gene, whereas color intensity reflects average expression level.

In our final analyses, we assessed the clinical relevance of our findings. We therefore examined Gal-3 expression in human testicular and epididymal tissues. Similar to the murine model, Gal-3 expression increased from the testis toward the proximal epididymal regions and reached the highest levels in the CD (Fig. 8A, B and Supplementary Fig. 12A). This pattern was further supported at the transcript level, with significantly higher *LGALS3* expression in CS and CD compared with the testis (Fig. 8C).

Consistent with the murine epididymis, a greater number of epithelial and interstitial cells expressing Gal-3 were observed in distal compared with proximal regions. In contrast to the murine testis, however, Sertoli cells in the human testis expressed Gal-3 (Fig. 8B and Supplementary Fig. 12A). Interestingly, in inflamed human testis from infertile patients, Gal-3⁺ immune cells were localized within damaged seminiferous tubules and the interstitial compartment (Fig. 8D and Supplementary Fig. 12B), consistent with the pattern observed in the murine model.

To comprehensively characterize the Gal-3 expression profile across the healthy adult human testis, we reanalyzed publicly available single cell RNA sequencing (sc-RNA seq) datasets from healthy donors of reproductive age (17–48 years; *n* = 26; Supplementary Table 4) [26–29]. The sc-RNA seq analysis revealed that in addition to Sertoli cells and immune cells (macrophages, T cells), *LGALS3* transcripts were also detected in other somatic cells, including Leydig, peritubular, smooth muscle and endothelial cells as well as to a lesser extent in germ cells (Fig. 8E, F and Supplementary Fig. 13). Together, these findings indicate that Gal-3 expression in immune cells is conserved across species, highlighting its potential importance in the regulation of inflammatory responses.

## Discussion

Gal-3 is a pleiotropic molecule with established roles in inflammation, fibrosis, tissue repair, and immune cell regulation across multiple organ systems [12–14, 30]. Within the male reproductive tract, emerging evidence suggests that Gal-3 contributes to immune regulation and tissue homeostasis, including roles in maintaining testicular macrophages and supporting germ cell survival [1, 21]. However, its spatial distribution across the testis and epididymis, and its functional role remain incompletely defined. In this study, we therefore investigated the role of Gal-3 in the testis and epididymis under both steady-state and inflammatory conditions, two organs that play pivotal roles in male reproductive and endocrine function.

We found that Gal-3 exhibits a tissue-specific localization pattern and a gradient of expression, which is lowest in the testis and increases towards the distal parts of the epididymis. In the testis, Gal-3 is expressed mainly in immune cells such as antigen-presenting cells (macrophages, dendritic cells), lymphoid cells (T cells, B cells), and germ cells (specifically early and late round spermatids as well as elongated spermatozoa). These observations are in line with previous studies showing a role for Gal-3 in maintaining testicular macrophages and supporting germ cell survival [21].

In the epididymis, we found that immune cells exhibit different levels of Gal-3 expression: lower in proximal regions and higher in distal regions. Epithelial cells also express varying levels of Gal-3 across regions, with the highest expression observed distally. This gradient expression pattern has also been reported in human and rat epididymis [1, 22]. The increasing expression of Gal-3 towards the epididymal duct suggests a potential role in sperm maturation and storage, as well as in the acquisition of fertilizing capacity. In addition, the higher levels of Gal-3 in immune cells in distal regions may reflect a role in immune surveillance against ascending pathogens.

While Gal-3 is expressed in several immune cell types in both the testis and the epididymis, we showed that its depletion led to changes in the balance between macrophages and T cells, resulting in increased T cell populations in the testis and cauda epididymidis. This finding is in line with previous reports [15, 18, 20, 31]. Gal-3 influences T cells by regulating several processes, including proliferation, apoptosis, activation, migration, and cytokine production [14, 18, 32]. In line with these regulatory roles, overexpression of Gal-3 reduces T cell proliferation and cytokine secretion, whereas its deletion promotes increased activity and proliferation [18, 20]. At the mechanistic level, Gal-3 has been shown to modulate T cell activation by interacting with adaptor proteins, restricting T cell receptor (TCR) mobility, increasing the activation threshold, and promoting TCR internalization at the immunological synapse [14, 18, 33, 34]. Consistent with these findings, T cells lacking Gal-3 display increased production of pro-inflammatory cytokines, including IFN-γ, TNF, IL-6, IL-2, and IL-4 [16, 33, 35, 36]. Accordingly, we observed that T cells obtained from *Lgals3*^*-/-*^ mice exhibited higher production of IFN-γ, TNF, and IL-17. Together, these findings support a role for Gal-3 as a regulator of T cell activity, acting to restrain excessive pro-inflammatory responses.

Fine-tuning of T cell responses by Gal-3 is not only intrinsically regulated, but is also orchestrated through interactions with antigen-presenting cells such as macrophages and dendritic cells, which can further influence T cell profiles and activity [33, 34]. While macrophage numbers were reduced in the *Lgals3*^*-/-*^ testis and CD epididymidis, macrophages in EAEO are known to adopt an inflammatory phenotype that promotes fibrosis and contributes to germ cell damage [7, 8, 37, 38]. In this context, we show that co-culture of T cells with BMDMs lacking Gal-3 further enhances IFN-γ production. In line with this, increased IFN-γ secretion has also been reported in T cells cultured with dendritic cells lacking Gal-3 [39], supporting the concept that antigen-presenting cells can functionally reprogram T cell responses.

Consistent with this, *Lgals3*^*−/−*^ macrophages cultured with T cells exhibited increased expression of genes associated with pro-inflammatory function and antigen presentation, which may further amplify T cell-mediated immune responses and contribute to tissue damage in EAEO. In addition, Gal-3 has been shown to promote polarization toward an anti-inflammatory macrophage phenotype, whereas its depletion skews macrophage profiles toward a more inflammatory state [40, 41].

Of note, within testicular macrophages in *Lgals3*^*−/−*^ EAEO, a subset expressing MHC class II was upregulated. IFN-γ is known to induce MHC class II expression in macrophages via the class II transactivator (*Ciita*) [42, 43], and we observe that *Ciita* expression is strongly increased in Gal-3-deficient EAEO testis. In accordance with this, pro-inflammatory mediators (e.g., *Tnf, Ccl2, Il1b, Inhba*) were also elevated.

Given that EAEO represents an experimental model of T cell–mediated autoimmune inflammation of the testis [44–48], these findings suggest that inflammatory and fibrotic processes are exacerbated in the absence of Gal-3. Elevated IFN-γ may contribute to this phenotype by promoting pro-inflammatory T cell responses, directly damaging germ cells, and synergizing with TNF to amplify testicular injury [44, 49–52].

The mechanistic actions of Gal-3 in inflammatory diseases remain highly context-dependent and, at times, controversial, varying across organs and disease settings. This may explain why deletion of Gal-3 can worsen disease outcomes in some contexts while ameliorating pathology in others, occasionally even within the same organ [12, 13, 15, 16, 20]. In our study, we show that mice lacking Gal-3 exhibit exacerbated inflammatory and fibrotic responses during EAEO, which uncover a previously unknown regulatory role of Gal-3 in T-cell dependent immune response and subsequent tissue damage during EAEO. In contrast, other studies has demonstrated that Gal-3 deficiency results in milder experimental autoimmune encephalomyelitis (EAE), suggesting a pathogenic role for Gal-3 in that context [16]. Further studies, consistent with our findings, report that Gal-3 deficiency is associated with increased IFN-γ production, promoting spontaneous germinal center formation and exacerbating autoimmune responses through autoantibody production [15]. Similarly, in autoimmune myocarditis, Gal-3 depletion increases IFN-γ production by CD4^+^ T cells, enhances IgG deposition, and worsens disease severity [17]. By contrast, in tumor models, loss of Gal-3 has been associated with increased T cell expansion and reduced anti-inflammatory macrophage populations, suggesting that Gal-3 may support tumor growth by promoting immune suppression [53].

To further elucidate the full immune profile of cells involved in the exacerbated immune responses in *Lgals3*-deficient testis and epididymis, additional high-throughput approaches, such as single-cell transcriptomic resolving macrophage and T cell heterogeneity, defining interaction networks, or identifying Gal-3–dependent signaling states, would be valuable. However, the extensive cellular damage and pronounced fibrotic remodeling observed in this model may limit the feasibility of such analyses. Nonetheless, the availability of the EAEO model is helpful to overcome the limitation of human samples. Future studies are needed to address the direct cell-specific function of Gal-3 using targeted depletion of Gal-3 to analyze their role in inflammatory responses and fibrotic remodeling.

In summary, our data show that Gal-3 acts as a regulatory molecule in the testis and epididymis, modulating T cell and macrophage responses, and that its absence aggravates tissue damage during EAEO. The enhanced damage observed is likely driven, at least in part, by increased IFN-γ production from Gal-3-deficient T cells. Despite its widely recognized role in promoting inflammation, our findings implicate Gal-3–dependent regulation of T cell activity as a key mechanism suppressing immune-mediated testicular damage. Moreover, our study advances the understanding of the mechanisms underlying immune-mediated male infertility and, more broadly identifies Gal-3 as an important contributor to testicular and epididymal physiology and immunology.

## Materials and methods

### Animals

Adult (10–12-week-old) C57BL/6 J mice were purchased from Charles River Laboratories (Sulzfeld, Germany). Homozygous *Lgals3*^*tm1Ftl*^ (*Lgals3*^*−/−*^) mice were bred on a C57BL/6 genetic background [21, 54]. *Zbtb46*^GFP^ mice were also used to localize dendritic cells. Animals were maintained under specific pathogen-free conditions (12 h light/dark cycle, 20–22 °C) and had access to water and food ad libitum. Age-matched male mice between 10-12 weeks were used for all experiments.

All experiments involving animals were conducted in strict accordance with the Guide for the Care and Use of Laboratory Animals and the German Animal Welfare Law. Animal experiments were performed in accordance with the recommendations of the local animal welfare ethics authorities (Regierungspraesidium Giessen, G 68/2021, Nr. 1057_GP).

### Induction of experimental autoimmune epididymo-orchitis (EAEO)

EAEO was induced in male *WT* and *Lgals3*^*−/−*^ mice as previously described [7–9]. Briefly, mice were immunized three times with testicular homogenate (TH) in complete Freund’s adjuvant (CFA; Sigma-Aldrich, St. Louis, USA). Injections were performed subcutaneously every 14 days, followed by an intraperitoneal injection of 100 ng *Bordetella pertussis* toxin (Calbiochem, Darmstadt, Germany) in Munõz buffer (25 mM Tris, 0.5 M NaCl, 0.017% Triton X-100, pH 7.6). Only TH-immunized *WT* mice exhibiting the pathological features of EAEO such as significantly reduced testis size and weight, an inhomogeneous testicular parenchyma, and erythema were included in the final analyses.

Adjuvant control animals received, using the same regimen, CFA mixed with 0.9% NaCl instead of TH. Age-matched untreated *WT* and *Lgals3*^*−/−*^ mice were also included. Animals were sacrificed 50 days after the first immunization using 5% isoflurane anesthesia followed by cervical dislocation.

The epididymides and testes were removed and either snap-frozen in liquid nitrogen, fixed in 10% PFA for cryosection staining, or fixed in Bouin’s solution for paraffin embedding. Fresh epididymides and testes were used for flow cytometric analysis.

The approximate sample size was determined a priori using statistical power analysis (G*Power software, version 3.1.9.7, Heinrich Heine University, Düsseldorf, Germany). Animals were randomly assigned to the experimental groups before the start of immunization procedure.

### Human patient cohorts and ethical approval

Testicular biopsies were obtained from the Giessen Testicular Biopsy Repository. Samples were either fixed in Bouin’s solution and embedded in paraffin or snap-frozen. Testicular surgeries were performed following the diagnosis of obstructive or non-obstructive azoospermia [55]. Biopsies were categorized as having impaired spermatogenesis accompanied by focal leukocytic infiltration (inflamed testis; n = 9) or intact spermatogenesis lacking signs of inflammation (control testis; *n* = 7) [7, 56–58].

Specimens of non-inflamed epididymis (*n* = 7), subdivided into the caput, corpus, and cauda regions, were retrieved from patients undergoing orchiectomy for testicular cancer at the Department of Urology, Pediatric Urology, and Andrology, Giessen, Germany (Giessen Testis Cancer Patient Cohort). Epididymal tissue samples were morphologically intact, non-inflamed, and without evidence of neoplastic infiltration. The study was approved by the Ethics Committee of the medical faculty of the Justus Liebig University, Giessen (AZ 26/11, 152/16). All patients provided written consent for surgery and histological examination for research purposes.

### Histological staining

Paraffin-embedded tissue sections from mouse and human tissues were stained using azo-carmine and aniline blue (Azan) staining [59].

### Immunofluorescence staining

Paraffin-embedded, Bouin-fixed tissue sections (5 µm) or 4% paraformaldehyde (PFA)-fixed frozen sections (20 µm) were used for immunofluorescence staining.

Paraffin-embedded sections were deparaffinized, rehydrated, washed in TBS, and subjected to antigen retrieval using either sodium citrate buffer (10 mM, pH 6.0) or proteinase K solution (0.6 units/mL in TE buffer, pH 8.0). Primary antibodies (Supplementary Table 1) were applied, followed by incubation with appropriate secondary antibodies. After washing, sections were treated with 0.3% Sudan Black in 70% ethanol for 5 min and mounted with ProLong Gold Antifade Mountant containing DAPI (Thermo Fisher Scientific). All antibodies were diluted in TBS.

PFA-fixed cryosections were air-dried before staining. After washing in TBS + 0.05% Tween, pH 7.6 (TBS-T), sections were permeabilized using 0.2% Triton X-100. Primary antibodies (Supplementary Table 1) were diluted in TBS-T, followed by incubation with appropriate secondary antibodies (Supplementary Table 1). Sections were then mounted with ProLong Gold Antifade Mountant containing DAPI. For negative controls, primary antibodies were omitted (Supplementary Fig.14).

Fluorescence images were acquired using a LSM 710 confocal laser scanning microscope (Carl Zeiss, Göttingen, Germany), a Stellaris 5 microscope (Leica Microsystems, Wetzlar, Germany), or a Leica Thunder wide-field fluorescence microscope (Leica Microsystems).

### High-dimensional flow cytometry

For flow cytometric analysis, single-cell suspensions from testis and epididymis were generated as previously described [57]. Briefly, collected epididymides, pooled from three mice, were divided into four regions and digested in PBS containing 1.5 mg/mL collagenase A (Roche Diagnostics, Mannheim, Germany) for 45 min at 37 °C to generate single-cell suspensions. The resulting cell suspension was passed through a 20 G needle and filtered through a 70 µm strainer. For the EAEO study, only the cauda epididymidis was used.

Decapsulated testes were digested in 1.2 mg/ml collagenase A and 15 U/ml DNase I (Roche Diagnostics) in a 34 °C water bath for 15 min. After washing, cells were centrifuged at 300×*g* for 10 min. Cells were stained with Zombie UV dye (BioLegend, San Diego, USA) for 15 min at room temperature (RT). After centrifugation, cells were incubated with FcR block (Miltenyi Biotec, Bergisch-Gladbach, Germany) for 10 min at 4 °C in Brilliant Stain Buffer (BD Biosciences, San Jose, USA) followed by incubation with appropriate antibodies (Supplementary Table 2) for 30 min at 4 °C.

Analysis was performed using a BD FACSymphony S6. Data were analyzed using FlowJo v10 (FlowJo LLC, Ashland, OR, USA). Samples were also downsampled to a defined number of immune cells, concatenated, and analyzed using the dimensionality reduction method t-SNE (t-distributed stochastic neighbor embedding).

### Generation of bone marrow-derived macrophages, T cell isolation, and co-culture

Bone marrow–derived macrophages (BMDMs) were generated as previously described [7]. Briefly, murine bone marrow progenitor cells were stimulated with recombinant murine macrophage colony-stimulating factor (M-CSF, 50 ng/mL; Miltenyi Biotec) in RPMI-1640 medium for 5 days. After 3 days of culture, the medium was changed. Splenic T cells were isolated by negative selection using the mouse Pan T Cell Isolation Kit II (Miltenyi Biotec) according to the manufacturer’s instructions, as previously described [7]. The isolated cells were cultured in 96-well plates in the presence of anti-CD3 (10 µg/mL; BioLegend, San Diego, USA) and anti-CD28 (1 µg/mL; BioLegend) antibodies for 2 days.

Subsequently, T cells were cultured alone or co-cultured with BMDMs for an additional 2 days. Cells were then stimulated with a cell stimulation cocktail (Thermo Fisher Scientific, Roskilde, Denmark) for 6 h. For the final 5 h of culture, brefeldin A (BioLegend) was added. T cells were then processed for flow cytometry staining, while BMDMs were collected for RNA isolation.

### Flow cytometry of cultured murine T cells

T cells obtained from in vitro culture were subjected to flow cytometry staining to assess cytokine production. Cells were stained with Fixable Viability Dye eFluor™ 450 (Thermo Fisher Scientific) for 30 min at room temperature (RT). Cells were then treated with FcR block for 10 min at 4 °C, followed by staining with appropriate cell surface antibodies for 30 min (Supplementary Table 2).

Before intracellular staining, cells were fixed and permeabilized using the Inside Stain Kit (Miltenyi Biotec). Data were acquired on a MACSQuant 10 flow cytometer and analyzed using FlowJo v10 software.

### RNA isolation and qRT-PCR

Total RNA was isolated from frozen mouse testes using the RNeasy Fibrous Tissue Mini Kit (Qiagen, Hilden, Germany) and from murine BMDMs using the RNeasy Mini Kit (Qiagen), according to the manufacturer’s instructions. RNA from human samples was isolated using QIAzol Lysis Reagent (Qiagen) following the manufacturer’s instructions. Contaminating genomic DNA was removed using the RNase-Free DNase Set (Qiagen). RNA from murine BMDMs (400 ng) and human tissues (1 µg) was reverse-transcribed as previously described [57]. Quantitative real-time polymerase chain reaction (qRT-PCR) was performed using iTaq Universal SYBR Green Supermix (Bio-Rad, Munich, Germany) on a CFX96 Touch real-time PCR detection system (Bio-Rad). Primer sequences are listed in Supplementary Table 3. Relative gene expression was analyzed using the 2^-ΔΔCt^ method [60].

### Library preparation and RNA sequencing (RNA-Seq)

Genome-wide gene expression profiling was performed by the Institute for Lung Health – Genomics and Bioinformatics, Justus Liebig University Giessen, Germany. Polyadenylated mRNA was isolated from 200 ng of total RNA per sample using the NEBNext® Poly(A) mRNA Magnetic Isolation Module (New England BioLabs). Sequencing libraries were constructed using the NEBNext® Ultra™ II Directional RNA Library Prep Kit for Illumina®, following the manufacturer’s instructions. Library quality control was assessed by capillary electrophoresis (4200 TapeStation, Agilent), and cDNA libraries were sequenced on an Illumina NovaSeq 6000 platform to generate 50 bp paired-end reads.

Raw sequencing data were demultiplexed and converted to FASTQ format using Illumina bcl2fastq software (v2.19.0.316). Downstream processing, including quality control, read filtering and trimming, alignment, and generation of gene-level count matrices, was performed using the nf-core RNA-seq v3.7 pipeline [61] implemented in Nextflow v23.04.03. Reads were aligned to the *Mus* musculus mm10 reference genome with gene annotations from Illumina’s iGenome repository (https://support.illumina.com/sequencing/sequencing_software/igenome.html). Default parameters were used in Docker mode. Subsequent analyses, including normalization and differential gene expression testing, were performed in R using DESeq2 [62]. Principal component analysis (PCA) was performed based on the 500 most variable genes. Heatmaps were generated using the ComplexHeatmap package [63]. Differentially expressed genes (DEGs) were ranked by adjusted *P* value, with the top 100 genes selected for downstream analyses. Pathway enrichment and gene set analyses were performed using clusterProfiler [64] against Gene Ontology (GO) and Reactome databases. Raw sequencing data and the processed gene expression matrix were deposited in the NCBI Gene Expression Omnibus under accession number GSE332990.

### Single-cell transcriptomics of the human testis

In this study, publicly available sc-RNA seq datasets were obtained from the Gene Expression Omnibus (GEO) repository under the accession numbers GSE254315, GSE182786, GSE124263, and GSE120508 [26–29]. Only samples derived from healthy individuals younger than 50 years of age were included in the analysis. Accordingly, 14 of the 25 available samples from GSE254315, four samples (GSM5536958, GSM5536959, GSM5536960, and GSM5536961) from GSE182786, two samples (GSM3526588 and GSM3526590) from GSE124263, and six samples (GSM3052917, GSM3052918, GSM3052919, GSM3052920, GSM3052921, and GSM3052922) from GSE120508 fullfilled the inclusion criteria and were included in the analysis (Supplementary Table 4). Downstream analysis was performed using Scanpy [65]. Low quality cells and outliers were filtered out based on the number of detected genes and total unique molecular identifier (UMI) counts using the 1st and 98th percentile thresholds. Cells with mitochondrial gene fraction exceeding 20% were additionally excluded. Following quality control, cells underwent downstream processing, including doublet detection using Scrublet, normalization, data integration using Harmony, and clustering. Clusters with high Scrublet doublet scores were subsequently removed. Cell clusters were manually annotated based on the expression of canonical marker genes (Supplementary Fig. 13). Macrophages and T cells were identified by the expression of *CD14*/ *C1QC*, and *CD3D/ CD3E*, respectively. Sertoli cells were identified based on *SOX9* and *PRND* expression, whereas Leydig cells were defined by *IGF1* and *DLK1* expression. Smooth muscle cells were characterized by *ACTA2* and *NOTCH3* expression, while peritubular cells were identified using *ACTA2* and *WFDC1* expression. Endothelial cells were annotated by *VWF* and *CD34* expression. Germ cell populations were classified according to developmental stage, including spermatogonia (*PIWIL4, ESX1, DMRT1, HMGA1*), spermatocytes (*SPAG6, SPATA8, PIWIL1*), round spermatids (*ACRV1, TEX29*), and elongating spermatids (*PRM3, TNP2, OAZ3*).

### Statistical analyses

Statistical analyses were performed using GraphPad Prism 10 (GraphPad Software, San Diego, USA). Data collection and analysis were performed without blinding to the experimental conditions. To ensure reproducibility, multiple biological replicates and independent experiments were conducted. Data were assessed for normality. Results are presented as mean ± standard error of the mean (SEM). Differences between two groups were evaluated using Student’s *t* test (for parametric data) or the Mann–Whitney test (for non-parametric data). For comparisons involving multiple groups, one-way or two-way ANOVA with Tukey’s post hoc test (for normally distributed data) or Kruskal–Wallis tests with Dunn’s post hoc comparisons (for non-parametric data) were applied. A *P* value < 0.05 was considered statistically significant.

## Supplementary information

Supplementary Table 1

Supplementary Table 2

Supplementary Table 3

Supplementary Table 4

Supplementary Fig. 1

Supplementary Fig. 2

Supplementary Fig. 3

Supplementary Fig. 4

Supplementary Fig. 5

Supplementary Fig. 6

Supplementary Fig. 7

Supplementary Fig. 8

Supplementary Fig. 9

Supplementary Fig. 10

Supplementary Fig. 11

Supplementary Fig. 12

Supplementary Fig. 13

Supplementary Fig. 14

## Data Availability

The generated datasets are available from the corresponding author upon reasonable request.
