# Supplementary material for: Galectin-3 restrains T cell-driven inflammation in the testis and epididymis
Source: Cell Death Dis. 2026 Sep 26;17(1):835. doi: 10.1038/s41419-026-09304-w (PMC13615981; doi:10.1038/s41419-026-09304-w)
Supplement: Supplementary file 1 [file 41419_2026_9304_MOESM1_ESM.pdf]

Supplementary Table 1. Primary and secondary antibodies as well as lectin for immunofluorescence staining.

**Primary antibodies**			
**Antibody**	**Company**	**Catalog No.**	**Dilution**
Goat polyclonal anti-Gal-3	R&D Systems, Minneapolis, USA	AF1197	1:30
Rabbit monoclonal anti-CD3ε	Cell Signaling Technology, Danvers, Massachusetts, USA	99940	1:50
Rabbit monoclonal anti-CD45	Cell Signaling Technology	D3F8Q	1:50
Rat monoclonal anti-F4/80	Cell Signaling Technology	71299	1:100
FITC anti-mouse anti-CD326 (Ep-CAM)	BioLegend, San Diego, USA	118207	1:50
Rabbit polyclonal anti-collagen I	Abcam, Cambridge, UK	ab21286	1:200
Rat monoclonal anti-GCNA1 (TRA98)	Abcam	ab82527	1:200
Rabbit polyclonal anti-gamma H2A.X (phospho S139)	Abcam	ab11174	1:300
Mouse monoclonal anti-CD45	Dianova GmbH, Hamburg, Germany	DLN-083890	1:30
Rat monoclonal anti-CD68	BioLegend	375602	1:20
**Lectin**	**Company**	**Catalog No.**	**Dilution**
Pure Arachis hypogaea lectin (PNA), FITC conjugated	EY Laboratories, San Mateo, USA	F-2301-1	1:200

Secondary Antibodies			
**Antibody**	**Company**	**Catalog No.**	**Dilution**
Goat anti-rat IgG (H+L) Alexa Fluor 546 conjugated	Invitrogen, Oregon, USA	A-11081	1:1000
Goat anti-rabbit IgG (H+L) Alexa Fluor 488 conjugated	Invitrogen	A-11034	1:1000
Goat anti-rabbit IgG (H+L) Alexa Fluor 546 conjugated	Invitrogen	A-11008	1:1000
Donkey anti-goat IgG (H+L) Cy3 conjugated	Jackson ImmunoResearch Labs, West Grove, USA	705-165-003	1:1000
Goat anti-rat IgG (H+L) Alexa Fluor 647 conjugated	Invitrogen	A21247	1:500
Goat anti-rabbit IgG (H+L) Alexa Fluor 750 conjugated	Invitrogen	A32733	1:500
Goat anti-mouse IgG F(ab)2 Alexa Fluor 488 conjugated	Jackson ImmunoResearch Labs	115-546-072	1:800
