# Supplementary material for: Galectin-3 restrains T cell-driven inflammation in the testis and epididymis
Source: Cell Death Dis. 2026 Sep 26;17(1):835. doi: 10.1038/s41419-026-09304-w (PMC13615981; doi:10.1038/s41419-026-09304-w)
Supplement: Supplementary file 2 [file 41419_2026_9304_MOESM2_ESM.pdf]

Supplementary Table 2. Antibodies flow cytometric analysis.

**Antibodies**	**Companies**	**Catalog No.**
**Antibodies for high dimensional flow cytometry**		
BD OptiBuild™ BUV563 rat anti-CD11b	BD Bioscience, Dümmer, Germany	741242
BD Horizon™ BUV805 rat anti-mouse CD45	BD Bioscience	568336
BD OptiBuild™ BUV615 hamster anti-mouse γδ T-cell receptor	BD Bioscience	751183
BD OptiBuild™ BUV661 rat anti-mouse Ly6G	BD Bioscience	741587
BD Horizon™ PE-CF594 rat anti-mouse CD192 (CCR2)	BD Bioscience	568804
Brilliant Violet 711™ rat anti-mouse CD4	BioLegend	100549
BV421 rat anti-mouse CD8a	BioLegend	100737
Brilliant Violet 785™ rat anti-mouse Ly-6C	BioLegend	128041
Brilliant Violet 605™ mouse anti-mouse NK-1.1	BioLegend	108739
Brilliant Violet 510™ rat anti-mouse I-A/I-E (MHC II)	BioLegend	107635
PE/Cyanine5 rat anti-mouse F4/80	BioLegend	123111
PE/Cyanine7 mouse anti-mouse CX3CR1	BioLegend	149015
Alexa Fluor® 700 rat anti-mouse CD206 (MMR)	BioLegend	141733

BD OptiBuild™ RB780 Rat Anti-Mouse CD19	BD Bioscience	755522
Brilliant Violet 650™ hamster anti-mouse TCR β chain	BioLegend	109251
Alexa Fluor® 488 rat anti-mouse/human Mac-2 (Galectin-3)	BioLegend	125410
APC hamster anti-mouse CD11c	BioLegend	117309
APC/Fire™ 750 rat anti-mouse CD3	BioLegend	100247
**Antibodies for assessment of cytokine production in cultured T cells**		
Recombinant monoclonal anti-mouse CD3 APC REAfinity™	Miltenyi Biotec, Bergisch Gladbach, Germany	130-122-943
Recombinant monoclonal anti-mouse CD4 VioBright FITC REAfinity™	Miltenyi Biotec	130-118-692
Recombinant monoclonal anti-mouse CD8α PE Vio770 REAfinity™	Miltenyi Biotec	130-119-123
Recombinant monoclonal anti-mouse IFN-γ PE REAfinity™	Miltenyi Biotec	130-117-502
PerCP/Cy5.5 rat anti-mouse TNF	BioLegend	506321
APC/Cyanine7 rat anti-mouse IL-17A	BioLegend	506939
