# Supplementary material for: Galectin-3 restrains T cell-driven inflammation in the testis and epididymis
Source: Cell Death Dis. 2026 Sep 26;17(1):835. doi: 10.1038/s41419-026-09304-w (PMC13615981; doi:10.1038/s41419-026-09304-w)
Supplement: Supplementary file 3 [file 41419_2026_9304_MOESM3_ESM.pdf]

Supplementary Table 3. Primers for qRT-PCR.

Gene	forward	reverse	Product size (bp)
Mouse			
Ccl2	AGCTGTAGTTTTTGTCACCAAGC	GTGCTGAAGACCTTAGGGCA	155
H2-Ab1	GTGGTGCTGATGGTGCTG	CCATGAACTGGTACACGAAATG	76
Hprt	CTGGTAAAAGGACCTC	CTGAAGTACTCATTATAGTCAAG	110
Il10	GCTCCTAGAGCTGCGGACTGC	TTTCCGATAAGGCTTGGCAACCCA	137
Il18	GACAGCCTGTGTTCGAGGATATG	TGTTCTTACAGGAGAGGGTAGAC	159
Il1a	CGAAGACTACAGTTCTGCCATT	GACGTTTCAGAGGTTCTCAGAG	126
Inhba	AGAACGGGTATGTGGAGATAGA	GACTCGGCAAAGGTGATGAT	97
Mrc1	CTTCGGGCCTTTGGAATAAT	TAGAAGAGCCCTTGGGTTCA	150
Nos2	CAGAGGACCCAGAGACAAGC	TGCTGAAACATTTCCTGTGC	299
Tnf	CCCTCCTGGCCAACGGCATG	TCGGGGCAGCCTTGTCCCTT	109
Human			

LGALS3	CAGAATTGCTTTAGATTTCCAA	TTATCCAGCTTTGTATTGCAA	108
RPL19	ATCCACAAGCTGAAGGCAGA	GCGTGCTTCCTTGGTCTTA	87
RPS27	GTGAAATGCCCAGGATGCTATA	TGTAGGCTGGCAGAGGACAG	99
