# Supplementary material for: Galectin-3 restrains T cell-driven inflammation in the testis and epididymis
Source: Cell Death Dis. 2026 Sep 26;17(1):835. doi: 10.1038/s41419-026-09304-w (PMC13615981; doi:10.1038/s41419-026-09304-w)
Supplement: Supplementary file 4 [file 41419_2026_9304_MOESM4_ESM.pdf]

Supplementary Table 4. Information on human testicular sc-RNA seq samples downloaded from NCBI's GEO data repository and corresponding accession numbers.

SampleID	GEO accession #	Dataset/ Refeerence	Age (Years)	Total Cells
GSM8037606	GSE254315	Cui et al., 2025	30	10814
GSM8037607	GSE254315	Cui et al., 2025	32	3685
GSM8037608	GSE254315	Cui et al., 2025	43	3676
GSM8037610	GSE254315	Cui et al., 2025	38	3357
GSM8037611	GSE254315	Cui et al., 2025	43	4002
GSM8037615	GSE254315	Cui et al., 2025	46	2781
GSM8037616	GSE254315	Cui et al., 2025	48	2698
GSM8037619	GSE254315	Cui et al., 2025	44	4905
GSM8037621	GSE254315	Cui et al., 2025	33	4246
GSM8037622	GSE254315	Cui et al., 2025	39	4082
GSM8037623	GSE254315	Cui et al., 2025	42	5690
GSM8037624	GSE254315	Cui et al., 2025	43	5532
GSM8037625	GSE254315	Cui et al., 2025	43	1756
GSM8037626	GSE254315	Cui et al., 2025	44	3484
GSM3052917	GSE120508	Guo et al., 2018	17	931
GSM3052918	GSE120508	Guo et al., 2018	17	1161
GSM3052919	GSE120508	Guo et al., 2018	24	864
GSM3052920	GSE120508	Guo et al., 2018	24	874
GSM3052921	GSE120508	Guo et al., 2018	25	1032
GSM3052922	GSE120508	Guo et al., 2018	25	1048
GSM5536958	GSE182786	Nie et al., 2022	17-22	3558
GSM5536959	GSE182786	Nie et al., 2022	17-22	5269
GSM5536960	GSE182786	Nie et al., 2022	17-22	3490
GSM5536961	GSE182786	Nie et al., 2022	17-22	4259
GSM3526588	GSE124263	Sohni et al., 2019	37	4684
GSM3526590	GSE124263	Sohni et al., 2019	42	5438
